# Analyzing breast cancer invasive disease event classification through explainable artificial intelligence

**DOI:** 10.3389/fmed.2023.1116354

**Published:** 2023-02-02

**Authors:** Raffaella Massafra, Annarita Fanizzi, Nicola Amoroso, Samantha Bove, Maria Colomba Comes, Domenico Pomarico, Vittorio Didonna, Sergio Diotaiuti, Luisa Galati, Francesco Giotta, Daniele La Forgia, Agnese Latorre, Angela Lombardi, Annalisa Nardone, Maria Irene Pastena, Cosmo Maurizio Ressa, Lucia Rinaldi, Pasquale Tamborra, Alfredo Zito, Angelo Virgilio Paradiso, Roberto Bellotti, Vito Lorusso

**Affiliations:** ^1^IRCCS Istituto Tumori “Giovanni Paolo II”, Bari, Italy; ^2^INFN, Sezione di Bari, Bari, Italy; ^3^Dipartimento di Farmacia-Scienze del Farmaco, Università degli Studi di Bari Aldo Moro, Bari, Italy; ^4^Dipartimento di Fisica, Università degli Studi di Bari Aldo Moro, Bari, Italy; ^5^International Agency for Research on Cancer, Lyon, France; ^6^Dipartimento di Ingegneria Elettrica e dell'Informazione, Politecnico di Bari, Bari, Italy

**Keywords:** invasive disease events, breast cancer, explainable AI, 10-year follow up, 5-year follow up

## Abstract

**Introduction:**

Recently, accurate machine learning and deep learning approaches have been dedicated to the investigation of breast cancer invasive disease events (IDEs), such as recurrence, contralateral and second cancers. However, such approaches are poorly interpretable.

**Methods:**

Thus, we designed an Explainable Artificial Intelligence (XAI) framework to investigate IDEs within a cohort of 486 breast cancer patients enrolled at IRCCS Istituto Tumori “Giovanni Paolo II” in Bari, Italy. Using Shapley values, we determined the IDE driving features according to two periods, often adopted in clinical practice, of 5 and 10 years from the first tumor diagnosis.

**Results:**

Age, tumor diameter, surgery type, and multiplicity are predominant within the 5-year frame, while therapy-related features, including hormone, chemotherapy schemes and lymphovascular invasion, dominate the 10-year IDE prediction. Estrogen Receptor (ER), proliferation marker Ki67 and metastatic lymph nodes affect both frames.

**Discussion:**

Thus, our framework aims at shortening the distance between AI and clinical practice

## Introduction

Although breast cancer is a heterogeneous disease showing different prognosis, the research achievement and public health policy on the diagnosis and management of cancer patients has improved survival worldwide (1). However, the next challenge is to avoid disease recurrence or the appearance of a second cancer (2, 3). In recent years, the application of machine learning (ML) and deep learning algorithms, often broadly referred as “artificial intelligence,” to support the diagnosis of breast cancer or to assist clinical decision making in breast cancer treatment, has been thoroughly investigated (4–6). Although these efforts led to different strategies (7, 8), a common aspect is the lack of transparent and easy ways to explain how a specific decision is achieved; this aspect is particularly important for the clinical applicability of a decision support system. To this aim, the novel paradigm of explainable artificial intelligence (XAI) (9–12) has been recently introduced. Thus, some explainable techniques with solid theoretical background, such as SHapley Additive exPlanations (SHAP), based on Shapley values computation (13), have been proposed to overcome the concept of artificial techniques as black boxes.

XAI applications could result critical for clinical studies more devoted to data analysis and characterization than clinical diagnosis; besides, it can be extremely relevant to explain the different performances observed (14). However, few research works addressed the design of an XAI framework for breast cancer (15–17).

Within this emerging pipeline, a plethora of predictive models based on ML have been developed to achieve a breast cancer recurrence prediction by solving a classification task (18–20) or focusing on survival (21). However, it is known that, despite not in common cases, anticancer drugs can cause second cancers, correlated with chemotherapy (22). In the adjuvant clinical trial setting for breast cancer, it has been proposed to adopt the term Invasive Disease-Event (IDE), to refer to composite events, such as local and distant recurrence, contralateral invasive breast cancers, second primary cancers (23). A survival model for IDE prediction and its variants have been recently proposed (24–26). These models were based on the exploitation of patients' characteristics related to demographics, diagnosis, pathology and therapy. However, these models based on ML algorithms resulted in weak explanations of the achieved prediction.

In this work, we focused on providing a statistically robust and explainable framework to identify the key-factors driving IDEs within two different periods: 5-years and 10-years. These frames are often adopted in clinical practice and also reported by others (2, 18).

A retrospective database of patients enrolled at the breast and clinic research center IRCCS Istituto Tumori “Giovanni Paolo II” in Bari (Italy) and containing features related to the primary tumor and the following therapy pathway was analyzed. State-of-the-art classifiers were used to assess, through a consensus strategy, the reliability of the features involved as well as the identification of the so-called confounding patients.

Then, we investigated the use of Shapley values to explain the patient classification in a simple and direct way. This explanation could be used by clinicians to make an informed decision on the reliability of model's prediction for each patient. Thus, this exploratory work opens the path to design fully automated explainable decision systems supporting clinical practice and shortening the gap between clinical practitioners and information provided by the artificial intelligence.

## Methods

### An XAI framework for IDE prediction

In this study, we propose an explainable machine learning framework to predict 5-year and 10-year invasive disease events. The analyzed data are provided by IRCCS Istituto Tumori “Giovanni Paolo II” in Bari (Italy) and refer to 486 patients registered for a first breast tumor diagnosis in the period 1995–2019. Patients who underwent a (i) neoadjuvant chemotherapy for breast cancer and/or (ii) had metastasis *ab initio* and/or (iii) had carcinoma *in situ* and/or (iv) followed up <10 years, with no relevant clinical events in the meantime, have not been included in this study. Breast cancer data were collected from the patients' medical records and comprised clinical and cytohistological outcomes. Moreover, we considered the therapy type and the related scheme for a comprehensive description of 28 features. Additional details are reported in Supplementary material (Data Collection section, Table S1).

**Figure 1:**
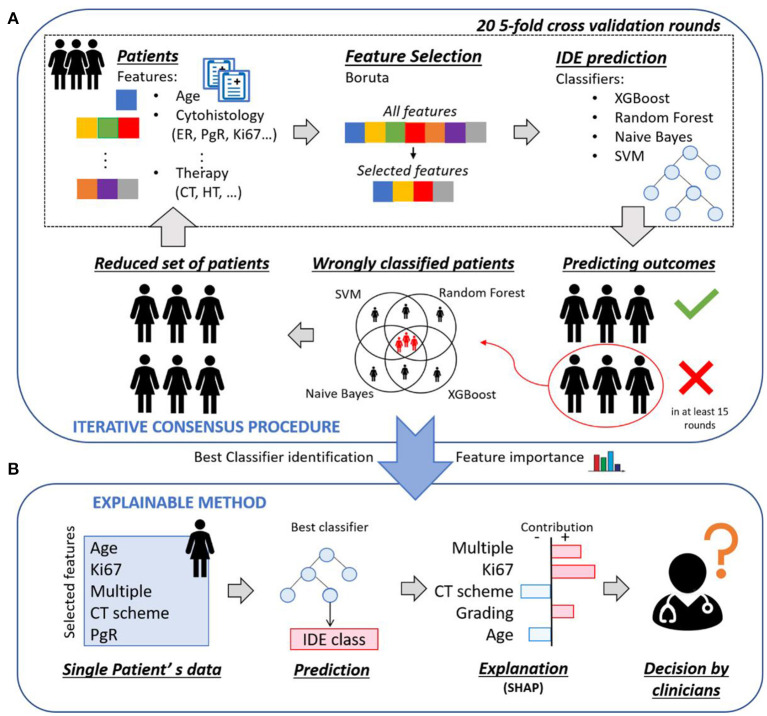
Overview of the proposed explainable framework for both 5-year and 10-year IDE prediction. **(A)** An iterative consensus procedure is used to define a reliable base of knowledge: first, a subset of features, that were relevant for the prediction outcome, were selected by means of Boruta technique; then, the IDE prediction were evaluated by means of four classifiers. The patients misclassified by all the four classifiers were identified and considered as confounding patients. **(B)** An explainable analysis determines individual patient classifications and outlines how features contributed to the decision of the model through the computation of Shapley values.

**Figure 2:**
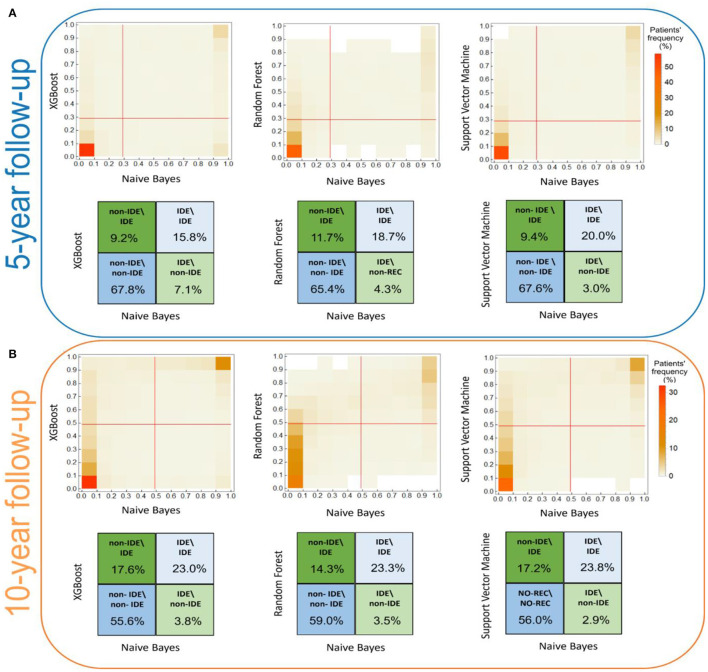
Score concordance maps and concordance tables for **(A)** the 5-year IDE prediction model and **(B)** the 10-year IDE prediction model. **(A, B)** The concordance maps (upper panel) represent the average histograms over 20 five-fold cross validation rounds of the classification score pairs, computed by the classifiers indicated in each axis. Red lines are referred to the score thresholds to assign a patient to the IDE class. **(A, B)** The concordance tables quantify the classification agreement (expressed in percentage values) among pairs of classifiers. Higher and lower intensity colors refer to higher and lower percentage values along the diagonals, respectively.

Figure 1 shows an overview of the implemented XAI framework, which consists of two main parts: (1) an iterative consensus procedure implemented to define a reliable base of knowledge as well as to identify the so-called confounding patients (Figure 1A); (2) an explainable analysis determined individual patient classifications and outlined how features contributed to the decision of the model (Figure 1B).

Starting from the breast clinical data of all the patients at disposal, a 20 five-fold cross validation rounds scheme was performed: first, a subset of features, that were relevant for the prediction outcome, was selected by means of Boruta technique (27); then, the IDE prediction was evaluated by means of four classifiers, that are Random Forest (28) (RF), Support Vector Machine (29) (SVM), XGBoost (30) (XGB) and Naive Bayes (31) (NB). For each classifier, the patients misclassified in at least 15 over 20 rounds were identified, where 15 over 20 corresponds to the third quartile upper bound. The patients misclassified by all the four classifiers were discarded and the remaining patients formed a reduced set of patients, to which the procedure could be re-applied. The entire process was indicated as iterative consensus procedure (Figure 1A). To our aim, two iterations of the consensus procedure were performed, since a trade-off between the classification concordance among classifiers and the number of discarded patients was reached (see Results section). The patients discarded after two iterations were indicated as confounding patients, while the remaining patients were indicated as included patients. The consensus between couple of classifiers in assigning labels to patients was measured by means of the Cohen's kappa (κ) coefficient (32). Specifically, κ coefficient measures the inter-reliability, i.e., the degree of agreement, among independent classifiers, which have addressed the same IDE prediction task, also taking into account the possibility of the agreement occurring by chance. Its values range in the interval values from [−1, 1]. A high value of κ coefficient means a great consensus between the analyzed classifiers. Different concordance degrees can be defined: if κ is <0 there is no concordance; if κ ranges in [0, 0.4], a poor concordance is shown; if κ belongs to [0.4, 0.6], it means there is a moderate concordance; if κ falls into [0.6, 0.8], a good concordance is achieved; if κ is included in [0.8, 1], a great concordance is observed.

After identifying the most occurring features in all the 20 rounds and the best performing classifier (see Results section), an explainable method, i.e., SHAP (13), based on Shapley values computation, was adopted to explain the contribution of each of the most important features to the decision of the best classifier over each included patient. An explanatory example is reported in Figure 1B: the model predicted that a patient belonged to the IDE class, and the corresponding Shapley values highlighted the features that led to prediction. Ki67, multiplicity and grading are portrayed as contributing to increase the score related to the IDE prediction, while chemotherapy (CT) scheme and age are evidence against it. Finally, a 20 five-fold cross-validation rounds scheme was utilized. Shapley values were also computed for confounding patients by employing all the included patients as training set and the confounding patients as test set.

### Feature selection, classification and performance evaluation

Within the iterative procedure, aML-based wrapper algorithm, known as Boruta (27), was used for feature selection. Boruta exploits Random Forest and iteratively compares the relevance of original attribute with that of their permuted copies, that are called shadow features. The real features that have significantly higher relevance than the shadow features are admitted as confirmed and are retained in the dataset; real features, that are significantly unimportant when compared to the shadow counterparts, are rejected and removed from the dataset. The algorithm stops when all the features are recorded as rejected or confirmed or when a priory set maximum number of iterations is reached. Here, the maximum number of iterations was set as 100.

The binary classification task (IDE vs. no-IDE) was then solved by training four machine learning classifiers, such as NB, SVM, RF, and XGB.

NB, SVM and RF are well-known classifiers. Here, the radial basis kernel function was employed for the SVM classifier. A configuration with 500 trees was adopted for the RF classifier. To avoid overfitting, a small number of observations per tree leaf, such as 5, has been fixed.

XGB is a decision-tree strategy-based ensemble algorithm deriving from the gradient boosting framework, where each individual tree is a sort of “weak predictor.” This algorithm has gained particular attention in the-state-of-the-art and developers given that it has yielded superior results in data mining competitions with a drastically reduced computational time (22). The tuning of several incorporated parameters guarantees its robustness to overfitting. Among them, we mention booster parameters related to the chosen booster, such as boosting learning rate (eta), number of boosted trees to fit (n_estimators), maximum tree depth for base learners (max_depth), minimum sum of instance weight needed in a child (min_child_weight), subsample ratio of columns (colsample_bytree), subsample ratio of the training instance (subsample). Similarly to Fu et al. (24), we set the parameters as follows: eta = 0.1, n_estimators = 100, max_depth = 5, min_child_weight = 2, subsample = 0.9, col-sample_bytree = 1.0. Learning task parameters related to the learning scenario also need to be defined, such as the objective function (objective) and the evaluation metric (eval_metric) that measures the performance of the objective function as a return value. We fixed objective = softprob, that returns predicted probability of each data point belonging to each class, and eval_metric = error, that is the binary classification error rate calculated as number of wrong cases over all cases.

The performances of the above-mentioned classifiers were evaluated in terms of Area Under the Curve (AUC) of the Receiver Operating Characteristic (ROC) curve and other standard metrics, such as accuracy, sensitivity, specificity, and F1-score. Each patient with a classification score exceeding a threshold, imposed as the ratio of the number of patients belonging to the IDE class over the total number of patients composing the dataset (33), has been assigned to the IDE class.

### An explainable algorithm: SHAP

In this manuscript, we adopted a cutting-edge local explanation algorithm, SHAP (13)^1^, to give an effective and faithful explanation of the most performing classifier's prediction. SHAP is a local model-agnostic approach, since it uses only the input and the output of a classifier. Specifically, regardless which concepts the classifier apprehends, it learns an interpretable linear model at local decision level, namely, it interprets the contributions of individual feature values on predictions referred to each test sample. The explanation of each feature is quantified in Shapley values. The Shapley absolute value is a measure of how much each feature contributes to the final prediction. Here, we want to give a general idea of how SHAP works. For a detailed mathematical underpinning, please refer to the works of Štrumbelj et al. (34) and Slack et al. (35).

Let *D* be a dataset of patients, *D* = [(**x**_1_, *s*_1_), (**x**_2_, *s*_2_), ..., (**x**_*N*_, *s*_*N*_)], where **x**_*i*_ represents the feature vector for the sample *i* and *s*_*i*_ the corresponding score to belong to the IDE class. The dataset can be split into a training set and a test set. Let *f* be a classifier and *f*(**x**_*i*_) the prediction component for the test instance *i* which corresponds to the IDE class. Our goal is to explain the contribution of each feature value to the *prediction difference*, i.e., the difference between the classifier prediction for the sample *i* and the expected prediction if all the feature values are not known, that is defined as the global average prediction over the training set. In **Figures 6**–**8** the expected prediction is reported as *base value*.

The core idea is to learn a linear explanation model *g* to explain *f*, where *g*∈*G*, the class of the linear models. The following optimization problem is solved:


(1) $$\arg L(f,g,\pi_{x^{\prime }})+\text{Ω}(g)$$

where Ω(*g*) is the complexity of the explanation model (measured as the number of non-zero weights) and $\pi_{x^{\prime }}$ is a proximity metric (local kernel) between inputs *x* and *x*′. Both Ω(*g*) and $\pi_{x^{\prime }}$ in Equation 1 are defined according to principles from cooperative game theory so that the resulting explanations satisfy some properties, that are local accuracy, missingness and consistency (34).

The loss function *L* is expressed as:


(2) $$\begin{array}{l} L\left(f,g,\pi_{x^{{}^{\prime }}}\right)=\underset{x^{{}^{\prime }}\in X^{{}^{\prime }}}{\sum }{\left[f\left(x^{{}^{\prime }}\right)-g\left(x^{{}^{\prime }}\right)\right]}^{2}\pi_{x^{{}^{\prime }}} \end{array}$$

where *X*′ represents the set of inputs within the neighborhood of *x*.

Basically, the explanation for *x* accurately approximates the behavior of the model within the neighborhood of *x*, while achieving lower complexity (35).

Finally, it is remarkable to note how such a method considerably differs from feature selection techniques. Feature selection methods aim to establish the relevance of each feature on a performance metric by using the training set. As result, a single final feature importance vector is obtained, whereas SHAP returns one feature importance vector per test instance. In this way, each patient is associated with a diverse feature importance vector.

## Results

### Consensus analysis

We performed two iterations of the *consensus procedure*, as described in Methods section, and removed 59 and 64 patients for the subsequent analyses for the 5-year and 10-year periods, respectively. Then, we evaluated the concordance of the different classification models and observed how after two iterations all the considered models converged to the same performance and, meantime, the largest number of available patients was preserved. Moreover, the number of the most important features remained stable for the following iterations. Firstly, we evaluated the consensus of the four models in terms of the pairwise κ coefficient, as reported in Table 1.

**Table 1:** Cohen's kappa (κ) coefficient computed for pairs of classifiers before and after the application of the iterative consensus procedure.

		**RF-SVM**	**SVM-XGB**	**RF-XGB**	**XGB-NB**	**RF-NB**	**SVM-NB**
5-year follow-up	Whole cohort (*n* = 486)	0.54 ± 0.13	0.67 ± 0.16	0.68 ± 0.16	0.31 ± 0.08	0.45 ± 0.13	0.37 ± 0.10
	Selected cohort (*n* = 427)	0.69 ± 0.16	0.71 ± 0.17	0.75 ± 0.17	0.53 ± 0.12	0.65 ± 0.15	0.56 ± 0.13
10-year follow-up	Whole cohort (*n* = 413)	0.42 ± 0.11	0.53 ± 0.13	0.66 ± 0.16	0.40 ± 0.10	0.26 ± 0.07	0.36 ± 0.09
	Selected cohort (*n* = 349)	0.64 ± 0.15	0.69 ± 0.16	0.70 ± 0.16	0.48 ± 0.12	0.49 ± 0.12	0.42 ± 0.10

All the κ-coefficients are averaged over 20 five-fold cross validation rounds. Standard deviations are also reported.

The consensus among the classifiers increased after two iterations. As example, the κ coefficients between XGB and RF passed from 0.68 to 0.75 for the 5-year predictive model. Figure 2 shows the concordance maps and the concordance tables for both time periods, in which one of the two classifiers is NB for all the pairs (i.e., XGB vs. NB, RF vs. NB, SVM vs. NB). Each concordance map represents the average histograms over 20 five-fold cross-validation rounds of the classification score pairs yielded by the classifiers associated with each axis. Red lines are referred to the score thresholds to assign a patient to the IDE class (0.29 and 0.49 for 5-year and 10-year follow-up models, respectively, see Methods section). We chose to outline the comparison against NB: although its global concordance with all the classifiers was increased by the consensus procedure (as reported in Table 1), its dissimilar behavior enabled the exclusion of a low number of patients.

Overall, the lowest agreement emerges for the IDE class, 15.8%, 18.7%, 20.0% for NB vs. XGB, NB vs. RF, NB vs. SVM for the 5-year predictive model, and 23.0%, 23.3%, 23.8% for NB vs. XGB, NB vs. RF, NB vs. SVM for the 10-year predictive model, respectively. Conversely, for the non-IDE class we found a better agreement: 67.8%, 65.4%, and 67.6% (compared to XGB, RF, and SVM, respectively, for the 5-year predictive model) and 55.6%, 59.0%, and 56.0% (compared to XGB, RF, and SVM, respectively, for the 10-year predictive model, respectively). Overall, the consensus between couples of classifiers is stronger for the 5-year model than the 10-year model.

Of note, since the misclassified patients correspond to patients wrongly classified by all the four classifiers, these patients are considered confounding in a model-independent way, in which they are indistinguishable with respect to the considered features. A total of 59 patients (corresponding to 12% of the initial number of patients of which 40 IDE and 19 non-IDE) for the 5-year prediction model, and 64 patients (corresponding to 12% of the initial number of patients of which 54 IDE and 10 non-IDE) for the 10-year prediction model have been excluded from further analysis. Among them, 18 patients (16 IDE and 2 non-IDE) are in common between the two models. For both the predictive models, the majority of the confounding patients belong to the IDE class.

### Classification performance at 5-year follow-up

**Figure 3:**
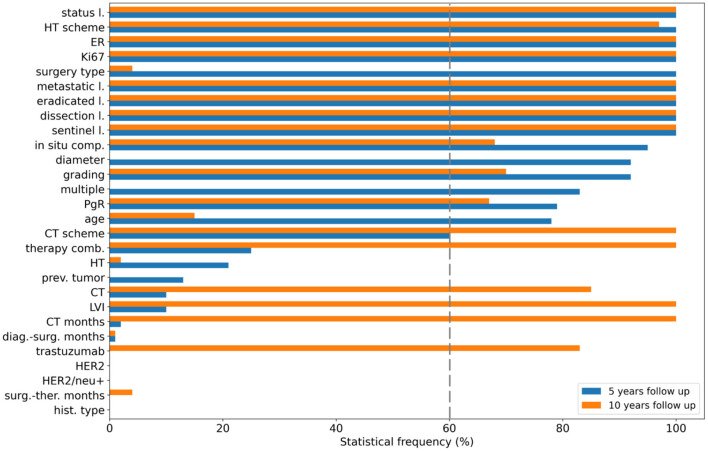
Feature selection. Statistical frequency of the features selected in nested cross-validation for the 5-year follow-up (blue bars) and the 10-year follow-up (orange bars). Features are ranked according to their importance with respect to the 5-year predictive model.

Here, we highlight the performances achieved by all the applied classifiers on the 427 patients at 5-year follow-up retained at the end of the consensus procedure. To ensure an unbiased evaluation, a nested feature importance by means of Boruta technique has been performed for both periods. Figure 3 shows the results for 5-year (blue bars) and 10-year (orange bars) follow-ups. For the 5-year period, 15 variables reached at least a 60% frequency, namely surgery type, age, diameter, multiple, i.e., multiplicity, ER, Ki67, number of eradicated lymph nodes, number of metastatic lymph nodes, lymph nodes status, sentinel lymph node, lymph node dissection, hormone therapy (HT) scheme, *in situ* component, PgR, grading and chemotherapy (CT) scheme (see Figure 3), and were then used for the subsequent explainable model.

**Figure 4:**
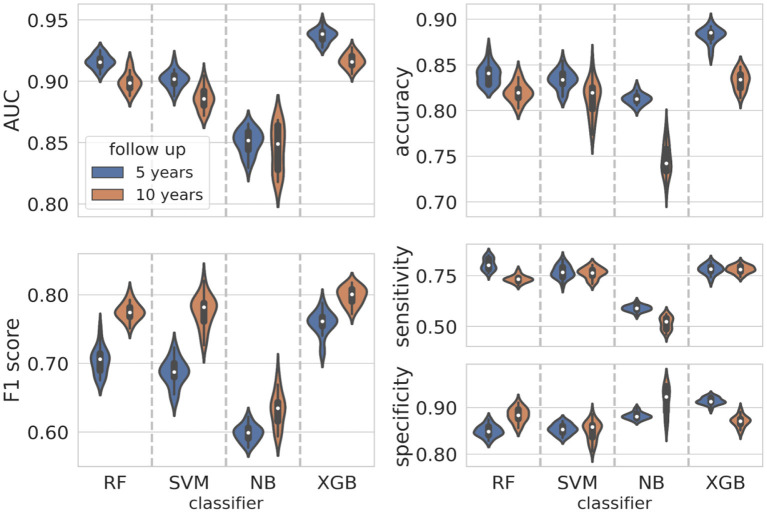
Classification performance distributions. Violin plots representing the classification performance distributions according to standard metrics such as AUC, accuracy, F1 score, sensitivity, and specificity (blue plots for the 5-year predictive model, orange plots for the 10-year predictive model). To have a better visualization of the performance distributions, the range on the y-axis is different in each panel.

The violin plots in Figure 4 represent the performance distributions of all the four classifiers expressed in terms of AUC, accuracy, F1 score, sensitivity and specificity (blue). The best cross-validation performance was obtained by the XGB classifier. The average cross-validation performance for all models and the related standard errors are reported in Supplementary Table S2. Overall, except for NB, all the models resulted well balanced in terms of sensitivity and specificity.

### Classification performance at 10-year follow-up

The initial dataset of patients at 10-year follow-up was reduced at 349 patients after applying the consensus procedure. In this case, the features selected with a frequency of more than 60% were 17, of which 12 features were in common with the 5-year model, namely ER, Ki67, eradicated lymph nodes, metastatic lymph nodes, lymph nodes status, sentinel lymph node, lymph node dissection, CT scheme, HT scheme, *in situ* component, PgR, grading; in addition, CT, CT months, trastuzumab, therapy combination and lymphovascular invasion (LVI) were also selected (orange bars in Figure 3). The performance distributions for all the classifiers are represented as orange violin plots in Figure 4.

As before, XGB globally outperformed all the other classifiers, whereas Naive Bayes confirms as the less performant classifier. The average values cross-validation values and the related uncertainties for AUC, accuracy, sensitivity, specificity and F1 score are reported in Supplementary Table S2.

### Explainability at global level

**Figure 5:**
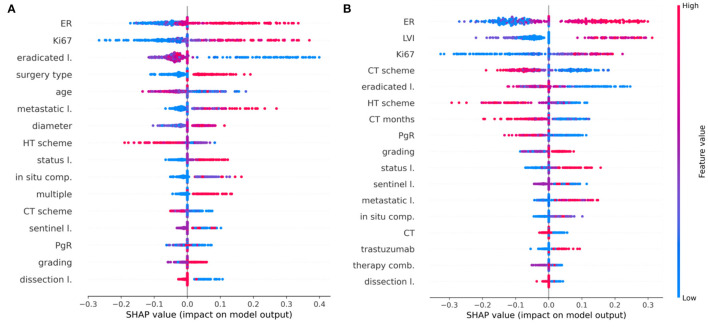
Shapley value distributions. Combination of feature importance with feature effects for **(A)** the 5-year IDE predictive model and **(B)** the 10-year IDE predictive model. Each point on the plot is a Shapley value for a feature and a patient. The color bar represents the value of the feature from low (in blue) to high (in red) for that instance. Integer values associated with categorical variables obey the following rules: *in situ* component (0, not present; 1, present, not typed; 2, G1; 3, G2; 4, G3), LVI (0, not present; 1, present, not typed; 2, focal; 3, extended), sentinel lymph node (0, negative; 1, not done; 2, positive), surgery type (1, quadrantectomy; 2, mastectomy), CT scheme (0, absent; 1, anthracycline + taxanes; 2, antracycline; 3, taxanes; 4, CMF; 5, other), HT scheme (0, absent; 1, Tamoxifen; 2, LHRHa; 3, Tamoxifen + LHRHa; 4, Aromatase Inhibitors; 5, Tamoxifen + Aromatase Inhibitors; 6, LHRHa+ Aromatase Inhibitors; 7, other), therapy combination (0, No; 1, HT; 2, CT; 3, CT + HT, 4, CT + trastuzumab, 5, CT + HT + trastuzumab).

The main purpose of this study is to outline how a machine learning algorithm achieves a certain decision, assigning a patient to the IDE class, when the follow-up is equal to 5 or 10 years after the first breast cancer diagnosis. Using the best classifier, XGB, on each test sample, we computed the Shapley values (see Methods section) using the Python SHAP package (see footnote 1). Only the important selected features (exceeding the 60% of occurrences) were evaluated. Figure 5 highlights which features mostly affected the decision scores (Figure 5A for the 5-year model, Figure 5B for the 10-year model, respectively).

Features are ranked in descending order according to their importance on the five-fold test sets over a specific round. Each point is the Shapley value for a feature and a sample. The relationship between a higher or lower feature value and a higher or lower prediction (classification score for the IDE class) also emerges.

Concerning the 5-year predictive model, ER, Ki67, lymph node status as well as tumor diameter, grading, multiplicity, and the number of metastatic lymph nodes positively contribute with IDE occurrence. Conversely, variables such as age and the number of eradicated lymph nodes show a negative contribution with respect to the IDE prediction score. Furthermore, the performed surgery type also contributes to the IDE occurrence (Figure 5A). Even for the 10-year predictive model, ER, Ki67, lymph node status as well as the number of eradicated lymph nodes, have a high impact on the IDE prediction score with a positive contribution for ER, Ki67 and lymph node status and a negative contribution for the number of eradicated lymph nodes. Moreover, the presence of LVI, low values of PgR expression and no previous hormonal therapy or chemotherapy, contributed to increase the IDE prediction score (Figure 5B). Chemotherapy schemes based on taxane alone or CMF (Cyclophosphamide, Methotrexate, Fluorouracil) tend to decrease the score.

### Explainability at local level

**Figure 6:**
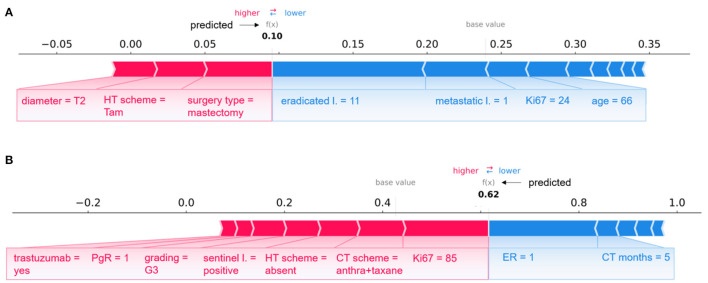
Examples of individual explanations for correctly classified patients. **(A)** A non-IDE patient for the 5-year predictive model and **(B)** an IDE patient for the 10-year predictive model. **(A, B)** Representation of the additive Shapley values: red color indicates a positive contributions, while blue a negative contributions.

**Figure 7:**
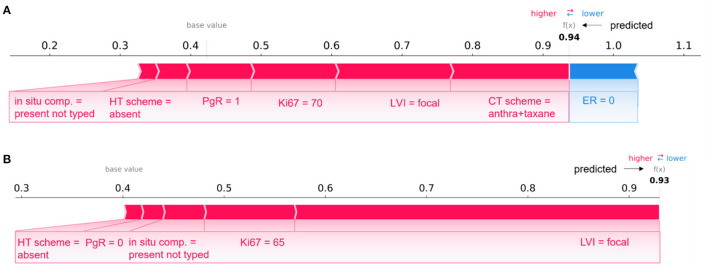
Comparison of individual explanations between correctly and wrongly classified patients. **(A)** Patient correctly classified (IDE class) and **(B)** patient wrongly classified (real class: non-IDE; predicted class: IDE) by the 10-year predictive model. **(A, B)** Representation of the additive Shapley values: red color indicates a positive contributions, while blue a negative contributions.

A global view of the Shapley value distributions, i.e., by considering all the patients retained after the consensus procedure, is given in Figure 5. However, Shapley values allow us to explain the contributions of individual features on predictions at local decision level, i.e., referred to each test sample separately. As example, Figures 6, 7 show some individual explanations. An explanation is a set of relative weights (Shapley values) for variables related to the first breast tumor and the subsequent performed therapy scheme: a feature either contribute to the prediction of the IDE event (positive sign in red) or do not (negative sign in blue). Specifically, each patient is associated with a diverse feature importance vector, in which the contribution of each feature (positive or negative) with respect to the classification score is computed in terms of Shapley value.

A pair of patients per class (non-IDE within 5 years, IDE within 10 years) correctly classified in a specific round is shown in Figure 6 (non-IDE within 5 years in Figure 6A, IDE within 10 years in Figure 6B).

Considering Figure 6A, XGB classifier returned a prediction score equal to 0.10. Thus, the patient was assigned to the non-IDE class. This can be related to the T2 tumor diameter, which contributes to the increase of the IDE prediction score. A similar effect on the prediction is given by the performed surgery type and HT scheme. In addition, an intermediate value for proliferative rate Ki67, a relatively high age and a low value of metastatic lymph nodes as well as a quite high number of eradicated lymph nodes go against the raise of the IDE prediction score.

The patient illustrated in Figure 6B is assigned to the IDE class with a prediction score equal to 0.62. In this case, a positive but low ER expression (equal to 1) together with the duration of the chemotherapy (5 months over the maximum duration of 6 months) contribute to decrease the IDE prediction score. By contrast, high values of Ki67 and grading, positive sentinel lymph nodes and a low value of PgR play a role in the increase of the prediction score. Of note, the therapies performed by the patient influence the growth of the IDE prediction score.

Figure 7 describes two patients whose classification score was predicted by means of the 10-year predictive model (XGB) after the consensus procedure.

The Figure 7A refers to patient correctly classified into the IDE class with a prediction score equal to 0.94; Figure 7B is related to a patient wrongly assigned to the IDE-class with a prediction score equal to 0.93. A patient was defined as correctly classified if XGB gave a right prediction in 19 over 20 rounds. As highlighted in the figure, the two patients share some variables, such as LVI, *in situ* component, HT scheme and Ki67, with equal or close values and the same positive impact on prediction. The PgR values were positive and negative for the two patients, respectively. However, they resulted both very close in the range of all the possible percentage values that PgR can assume. The two patients have also in common the ER value, but it contributes only to the prediction of the patient in Figure 7A. It can be justified since Shapley values attribute a weight to a feature when the feature is considered not alone but in cooperative relationship with all other features that can assume diverse values between the two patients. Indeed, the two patients differ, for example, for a diverse CT scheme (antra + taxane for the corrected classified patient, antra for the wrongly classified patient), a diverse surgery type (mastectomy for the corrected classified patient, quadrantectomy for the wrongly classified patient), a diverse age (equal to 60 and 34, respectively), and multiplicity (yes and no, respectively).

Overall, despite belonging to opposite classes (IDE and non-IDE, respectively), the two patients have in common some clinical variable values which contribute equally to the final classification.

### Confounding patients

As reported in the previous paragraph, an example of a couple of patients belonging to opposite classes (IDE vs. non-IDE) with similar clinical feature values and contributions to the final predictions has been discussed. Such a condition is more evident for confounding patients.

Overall, the average Shapley values related to correctly classified patients of a class (Supplementary Figures S1A, S2A) have the same sign and similar magnitude of the average Shapley values over the confounding patients belonging to the opposite class (Supplementary Figures S1B, S2B).

**Figure 8:**
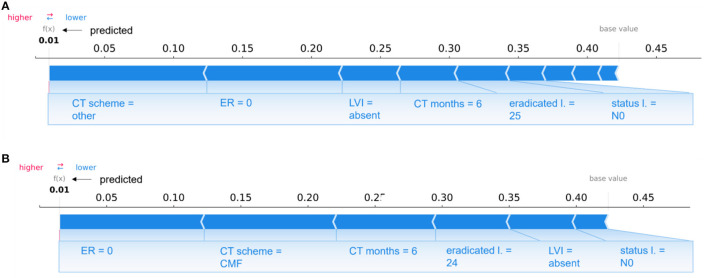
Comparison of individual explanations between a patient correctly classified and confounding patient. **(A)** Correctly classified patient (non-IDE class) and **(B)** wrongly classified patient (real class: IDE; predicted class: non-IDE) by the 10-year predictive model. **(A, B)** Representation of the additive Shapley values: red color indicates a positive contributions, while blue a negative contributions.

Specifically, in Figure 8, individual explanations of a couple of patients are shown: Figure 8A depicts the case of a patient correctly classified by the 10-year predictive model in the non-IDE class with a prediction score of 0.01; Figure 8B shows the case of a confounding patient belonging to the IDE-class but classified in the non-IDE class with a prediction score of 0.01.

The two patients share some variables, such as LVI, lymph node status, the duration of chemotherapy (CT months) and the number of eradicated lymph nodes, with equal or close values and the same influence on the prediction. The two patients have other similar features, such as PgR, ER, Ki67, *in situ* component, LVI, multiplicity, and age. None of the two patients underwent hormone therapy and the sentinel lymph node procedure since their surgery was before 2005. Both patients had a quadrantectomy surgery and a lymph nodes dissection with the same number of eradicated lymph nodes. However, they are characterized by two different CT schemes (other and CMF, respectively), that give the same sign contribution (negative) to the prediction. A diverse grading value also occurs (2 and 3, respectively).

## Discussion

In this work, we extended the application of an XAI framework to classification models for predicting 5-year and 10-year invasive disease events, respectively. The predictive models were obtained by four classifiers, namely, SVM, RF, NB, and XGB, analyzing characteristics strictly related to the first breast tumor and to the therapy scheme. The implementation of an iterative consensus procedure removed confounding patients resulting in high performing predictive models: the best classifier, namely, XGB, reached median AUC values equal to 93.7% and 91.7% for the 5-year and 10-year IDE predictions, respectively. Once reliable predictive models were obtained, an XAI method was implemented. Thus, we investigated the impact of the most important features on the IDE prediction score for each patient. The contributions of the interacting features were investigated by means of the Shapley values.

The study design was based on 5-year and 10-year IDE predictive models. Breast cancer relapse rates are commonly highest in the first 5 years (2). The adjuvant endocrine treatment for 5 years has become the standard of care. Nevertheless, the need to predict probabilities for later evidence of IDE is crucial to define long-term hormone treatments and personalized follow-up programs up to 10 years (36). The long-term therapy effect emerges from our analysis. CT and HT scheme result to be more important features for the 10-year prediction than the 5-year prediction (Figures 3, 5), whereas other features related to therapy, such as CT, CT months, therapy combination, and trastuzumab acquired importance only for the 10-year prediction. We found that undergoing HT therapy pathways, such as Tamoxifen, LHRHa, Tamoxifen +LHRHa, or CT planning based on taxane alone or CMF and a longer period of CT duration may reduce the risk of IDE.

In both 5-year and 10-year predictions, a primary role is played by the ER expression: a higher level of ER is associated with a higher risk of IDE. Patients with higher levels of ER did not undergo CT but were treated with a mild therapeutic plan consisting in HT, thus inducing a higher risk of IDEs. However, according to literature data, higher levels of ER expression are usually considered as a positive prognostic factor (37) for recurrence but, meantime, patients with ER-positive breast cancer maintain a significant recurrence rate during extended follow-up (38). The PgR expression resulted important for the 10-year prediction, showing a more marked negative contribution with IDE risk in this case. A lower PgR expression retains cancer cells in a “silent” mode during the HT within the first 5-years, after which they resume the replication phenomenon (39). Conversely to ER and PgR, the HER2 feature is not selected among the relevant features because it has a very lower variance (as reported in Table 1). The lymphovascular invasion does not appear as an important feature for the 5-year prediction, whereas it plays a key role for the 10-year prediction (Figures 3, 5) by gaining a positive contribution to IDE prediction especially when appearing in a focal or extended type (40) (Figure 5).

The Ki67 labeling index is recognized as a strong prognostic indicator of IDE occurrence for both predictive models. In the 5-year prediction, younger people (lower values of the age variable) with higher values of Ki67 are those with an increased IDE risk (41, 42) (Figure 5). Also, features, such as grading and lymph nodes status, are associated with a higher risk of IDE for both predictions (Figure 5). Similar findings have been extensively confirmed in the state-of-the-art for risk recurrence predictions (43, 44).

Concerning the 5-year prognostic model, IDEs are predominantly associated with aggressive tumors as measured by the tumor diameter, surgery type and multiplicity among the most important features. This observation is supported by the increased importance of those symptoms signaling metastatic phenomena, corresponding to the number of metastatic lymph nodes. Conversely, in both predictions, the lymph nodes dissection (as well as the number of eradicated lymph nodes and the not-done sentinel lymph node) represent a positive prognostic factor because it limits the persistence of the metastatic lymph nodes.

Finally, the presence of the *in situ* component emerges as an important feature considering both predictions. However, it is not accepted as a prognostic factor, but the *in situ* component has been recently recognized as a significant risk factor for intramammary recurrence (37).

Beyond the global evaluation of feature importance and impact on IDE prediction, the computation of Shapley values provides an explanation of a ML algorithm for each analyzed patient. Hence, a feature importance vector was returned for each patient. The weight of a feature expresses the contribution of that feature in interaction with the others to the ML algorithm's decision. Clinicians could use the prediction and the corresponding explanation to evaluate their reliability and make personalized treatment and care plans.

This study has some limitations. First, the presence of missing data which could introduce bias and affect the prediction efficiency. However, we included patients with missing data, due to the fact that is very common to have missing feature values since they could not be found in medical records. Then, a further limitation is represented by the non-involvement of external validation data. Nevertheless, in this work, our main goal was to give a detailed analysis of IDE prediction task through XAI, not providing a predictive model which could be effectively used in clinical practice. In addition, another limitation consists in the heterogeneous sample population under analysis. Specifically, the period of the first tumor diagnosis was over 20 years (from 1995 up to 2019), in which several pharmacological treatments have been introduced [53, 54]. Recently, schemes on sequential therapy with anthracycline and taxane (AC/EC/FEC × 3–4 cycles followed by taxane) or their combination (TAC/TEC) have been introduced into adjuvant clinical practice. These treatment regimens, involving the addition of the taxane, result in a reduction of 16% and 14% of the risk of recurrence and death, respectively. This means a significant gain of 4.6 and 3.2% in disease free survival and overall survival, respectively (45, 46). Furthermore, the addition of Trastuzumab for one year to CT with anthracycline and/or taxane has dramatically changed the natural history of BC HER2 neu + as shown by four randomized adjuvant studies, such as HERA, NSABP B-31, NCCTG N9831 and BCIRG 006. A broader and more homogeneous population, considering the period preceding the first tumor diagnosis should be studied in future evaluations. Nevertheless, since first-generation drugs, such as CMF, are still used (even if more rarely) in clinical practice together with new generation drugs, the predictive system also recognizes the effect of the first-generation drugs.

The focus of our study has been to evaluate and explain the role of the solely features related to the first tumor and the following therapy pathway on IDE prediction through an XAI framework. However, patients with the same primary tumor characteristics could respond to treatment in different ways (confounding patients) depending on other factors. Such an XAI framework explained *a posteriori* why the confounding patients were excluded. The Shapley values of excluded patients belonging to a class have the same behavior to Shapley values of the correctly classified patients belonging to the opposite class. This can be considered an exploratory study that contributes to design an effective clinical support tool which can be applied by medical experts, as additional tool to drive their choices with respect to tailored therapy pathways associated with the lower risk of an invasive disease event. Our future challenge is to design a system that integrates IDE prediction with *a priori* identification of confounding patients, thus allowing an effective application in clinical practice. Other variables related to demographics, quality of life, cardiology and hematology data will be included in future extensions of the analysis in order to investigate their contribution on IDE prediction for a possible discrimination of confounding cases. A complementary tool that is able to distinguish the specific typology of the invasive disease event (recurrence, contralateral breast cancer and second cancer) could be developed to evaluate the effectiveness of diverse treatment planning.

In conclusion, this work represents the first effort in integrating XAI in predictive models to give an IDE prediction. The definition of a potential XAI-clinical decision support system may emphasize the interplay between clinical experts and medical artificial intelligence. Indeed, the possibility to explain predictions can have a high impact in clinical practice since practitioners can effectively use reliable ML approaches. Furthermore, the possibility to obtain an explanation on the prediction for each individual patient would be beneficial to create “personalized” tools for clinicians to contribute to optimal selection of treatment and therapeutic options.

## Code availability

We made use of open-source software to conduct our experiments: the iterative consensus procedure was implemented using the R Software (v. 4.1.1, R Foundation for Statistical Computing, http://www.r-project.org/), while the explainable method was performed in Python (v.3.6.5). The “Boruta” R-package was used for implementing the Boruta technique; the “caret” R-package was used for implementing the classifiers; the “SHAP” Python-package was used for implementing the Shapley values computation and visualization (https://shap-lrjball.readthedocs.io/en/latest). We have described all the implementation details in the Methods section to allow for independent replication.

## Data availability statement

The raw data supporting the conclusions of this article will be made available by the authors, without undue reservation.

## Ethics statement

Ethical review and approval was not required for the study on human participants in accordance with the local legislation and institutional requirements. Written informed consent for participation was not required for this study in accordance with the national legislation and the institutional requirements.

## Author contributions

Conceptualization and supervision: RM, AF, and NA. Methodology and writing—original draft preparation: RM, AF, NA, MC, and DP. Software and validation: MC and DP. Formal analysis: RM, AF, NA, MC, DP, and AP. Resources: VL, RM, FG, and AZ. Data curation: RM, AF, SB, MC, and DP. Writing—review and editing: RM, AF, NA, SB, MC, DP, VD, SD, FG, DL, LG, ALa, ALo, AN, MP, CR, LR, PT, AZ, AP, RB, and VL. All authors have read and agreed to the published version of the manuscript.

## Footnotes

^1^ https://shap.readthedocs.io/en/latest/

## Author disclaimer

The authors affiliated to Istituto Tumori “Giovanni Paolo II,” IRCCS, Bari are responsible for the views expressed in this article, which do not necessarily represent the ones of the Institute. Where authors are identified as personnel of the International Agency for Research on Cancer/World Health Organization, the authors alone are responsible for the views expressed in this article and they do not necessarily represent the decisions, policy, or views of the International Agency for Research on Cancer/World Health Organization.
