# Supplementary material for: Analyzing breast cancer invasive disease event classification through explainable artificial intelligence
Source: Front Med (Lausanne). 2023 Feb 2;10:1116354. doi: 10.3389/fmed.2023.1116354 (PMC9932275; doi:10.3389/fmed.2023.1116354)
Supplement: Supplementary file 1 [file Data_Sheet_1.docx]

**Supplementary materials**

### Data Collection

Breast cancer data were collected from the patients’ medical records and comprised clinical and cytohistological outcomes, such as age at diagnosis (abbr. age), presence of previous tumors (abbr. prev. tumor, values: Yes/No), tumor diameter (abbr. diameter, values: T1a, T1b, T1c, T2, T3, T4), multiplicity (abbr. multiple, values: Yes/No) histological subtype (abbr. hyst. type, values: ductal, lobular, other), type of performed surgery (abbr. surgery, values: quadrantectomy/mastectomy), estrogen receptor (abbr. ER, % value), progesterone receptor (abbr. PgR, % value), cellular marker for proliferation (abbr. Ki67, % value), histological grade (abbr. grading, values according to Elston–Ellis scale: G1, G2, G3), human epidermal growth factor receptor-2 (abbr. HER2, value: Pos/Neg), HER2 score (abbr. HER2/neu+, values: 0,1,2,3), in situ component (abbr. in situ comp., values: absent, present but not typed, G1, G2, G3), lymphovascular invasion (abbr. LVI, values: absent, present but not typed, focal, extensive), lymph nodes status (abbr. status l., values: N0, N1, N2, N3), sentinel lymph nodes (abbr. sentinel l., values: Negative/Not done/ Positive), lymphadenectomy (abbr. dissection l., values: Yes/No), the number of eradicated lymph nodes (abbr. eradicated l.), the number of metastatic lymph nodes (abbr. metastatic l.). For those patients who were affected by bilateral or multiple tumours, we attributed the data referred to the greatest receptor expression.

Data related to the undertaken therapy type and the relative scheme were also included: chemotherapy (abbr. CT, values: Yes/No), hormone therapy (abbr. HT, values: Yes/No), trastuzumab (values: Yes/No), CT scheme (values: absent, Anthracycline (Anthra) + taxane, Anthra, taxane, CMF, other), HT scheme (values: absent, Tamoxifen (Tam), luteinizing hormone-releasing hormone analogues (LHRHa), Tam + LHRHa, aromatase inhibitor (AI), Tam + AI, LHRHa + AI, other), therapy combination (abbr. ther. comb, values: No, HT, CT, CT + HT, CT + trastuzumab, CT + HT + trastuzumab). Moreover, some ‘temporal’ variables were involved: CT duration expressed in months (abbr. CT months), time elapsed between the year of the first tumour diagnosis and surgery expressed in months (abbr. diag. - surg. months) and time elapsed between surgery and therapy initiation expressed in months (abbr. surg.- ther. months). Finally, a set of 28 features related to the primary breast tumour and the underwent therapy pathway was made up.

All these data materials were used to design a prognosis model to predict 5-year Invasive Disease Events (IDEs) following a first infiltrating breast cancer, which include local recurrence, the appearance of distant visceral and soft tissue metastases, contralateral invasive breast cancer or a second primary tumour18. A binary classification task was then formulated. In the following, IDE indicates the class of patients for whom an event occurs within 5 years from the first tumour diagnosis date (141 patients out of which 111 recurrence, 21 contralateral tumours and 9 second tumours); non-IDE class denotes those patients with at least 10-year follow-up and without an event within that timeframe (345 patients). A second prognosis model to predict 10-year IDEs (202 IDE patients, 211 non-IDE patients with at least 14-year follow up) was also developed. Table S1 summarizes the characteristics of collected data also underling missing data. Before the implementation of the consensus iterative procedure, missing clinical attribute values were treated according to a proximity technique. Firstly, each feature was rescaled by the maximum value. Then, given a patient with at least one missing clinical variable, a feature vector with only features without missing data was defined. Meanwhile, for each patient with complete data (198 in total), a feature vector with the same features of the given patient was constructed. The Euclidean distance between the feature vector of the given patient and the feature vectors of the patients with complete data was computed. Then, the missing values were replaced with the corresponding values of the patient with complete data whose feature vector had minimum distance from the feature vector of the given patient. The procedure was repeated for each patient with missing data.

**Supplementary Figures**


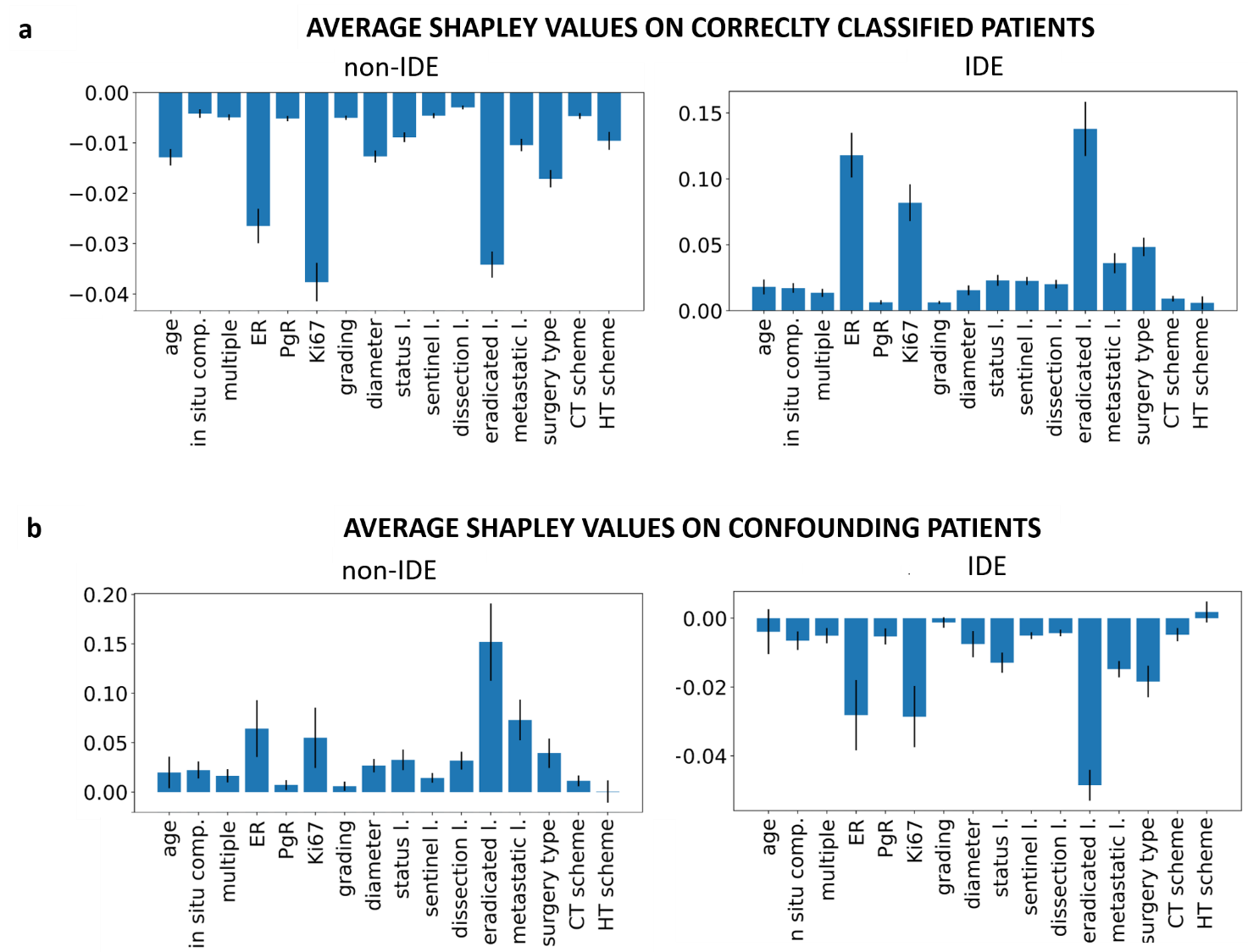


**Fig. S1. Comparison of average Shapley values for the 5-year predictive model**. The values are computed over **a)** the correctly classified patients and **b)** the confounding patients. For what concerns correctly classified patients, by averaging all the Shapley values related to one patient over the rounds, a vector containing Shapley values per patient was firstly constructed. One component was the average Shapley value for one feature. Then, after dividing the correctly classified patients in the two classes (IDE and non-IDE), a single Shapley value vector was crafted per class, where one component was the average contribution of one feature over all the correctly classified patients in that class, respectively. For what concern excluded patients, instead, Shapley values were computed by using the patients retained after the consensus procedure as training set and excluded patients as test set. Then, the average of Shapley values per feature was computed over all the excluded patients.


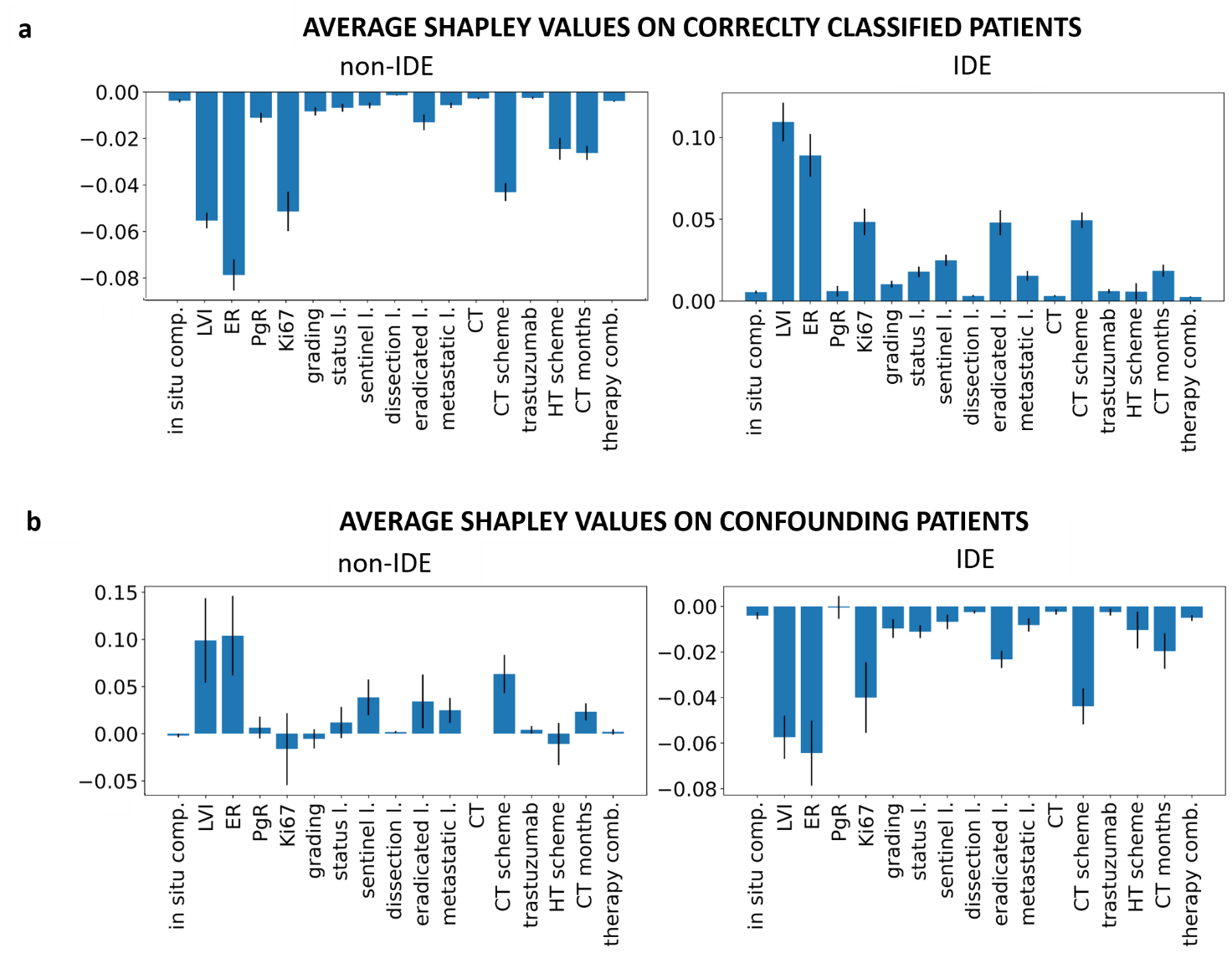


**Fig. S2. Comparison of average Shapley values for the 10-year predictive model.** The values are computed over **a)** the correctly classified patients and **b)** the confounding patients.

**Supplementary tables**

**Table S1. Observed patients’ statistics according to the considered features.** Absolute and percentage counts are reported in round brackets. For age, ER, PgR, Ki67, eradicated l. and metastatic l., CT months, diag. – surg. months, surg. – ther. months, the median value and first (q_1_) and third (q_3_) quartiles of the distribution are indicated in squared brackets. The number of missing values (NA) is also specified.

| **Features** | **Distribution** | **Features** | **Distribution** |
| --- | --- | --- | --- |
| **Overall** | 486; 100 % | N2 (abs.; %) | 36; 7.4 % |
| **Age** |  | N3 (abs.; %) | 19; 3.9 % |
| Median; [$q_{1},$ $q_{3}$] | 52 [45,61] | NA (abs.; %) | 10; 2.1 % |
| **Previous tumor** |  | **Lymphadenectomy** |  |
| Yes (abs.; %) | 15; 3.0 % | No (abs.; %) | 47; 9.7 % |
| No (abs.; %) | 471; 97.0 % | Yes (abs.; %) | 428; 88.0 % |
| **Tumor Diameter** |  | NA (abs.; %) | 11; 2.3 % |
| T1a (abs.; %) | 16; 3.3% | **Sentinel Lymph Node** |  |
| T1b (abs.; %) | 40; 8.2 % | Not Done (abs.; %) | 402; 82.7 % |
| T1c (abs.; %) | 207; 42.6 % | Positive (abs.; %) | 30; 6.2 % |
| T2 (abs.; %) | 174; 35.8 % | Negative (abs.; %) | 48; 9.9 % |
| T3 (abs.; %) | 12; 2.5 % | NA (abs.; %) | 6; 1.2 % |
| T4 (abs.; %) | 21; 4.3 % | **Eradicated lymph nodes** |  |
| NA (abs.; %) | 16; 3.3 % | Median; [$q_{1},$ $q_{3}$] | 19 [14,24] |
| **Multiplicity** |  | NA (abs.; %) | 18; 3.7% |
| Yes (abs.; %) | 99; 20.4 % | **Metastatic lymph nodes** |  |
| No (abs.; %) | 385; 79.2 % | Median; [$q_{1},$ $q_{3}$] | 0 [0,2] |
| NA (abs.; %) | 2; 0.4 % | NA (abs.; %) | 29; 6.0 % |
| **Histologic Subtype** |  | **Chemotherapy** |  |
| Ductal (abs.; %) | 430; 88.5 % | No (abs.; %) | 136; 28.0 % |
| Lobular (abs.; %) | 39; 8.0 % | Yes (abs.; %) | 348; 71.6 % |
| Other (abs.; %) | 17; 3.5 % | NA (abs.; %) | 2; 0.4 % |
| **Type of Surgery** |  | **Hormonotherapy** |  |
| Quadrantectomy (abs.; %) | 311; 64.0 % | No (abs.; %) | 144; 29.6 % |
| Mastectomy (abs.; %) | 175; 36.0 % | Yes (abs.; %) | 340; 70. 0 % |
| **ER** |  | NA (abs.; %) | 2; 0.4 % |
| Median; [$q_{1},$ $q_{3}$] | 45 [0,80] | **Trastuzumab** |  |
| NA (abs.; %) | 5; 1.0% | No (abs.; %) | 422; 86.8 % |
| **PgR** |  | Yes (abs.; %) | 63; 13.0 % |
| Median; [$q_{1},$ $q_{3}$] | 20 [0,70] | NA (abs.; %) | 1; 0.2 % |
| NA (abs.; %) | 6; 1.2 % | **CT scheme** |  |
| **Ki67** |  | Absent (abs.; %) | 136; 28.0 % |
| Median; [$q_{1},$ $q_{3}$] | 20 [10,40] | Anthra. + taxane (abs.; %) | 75; 15.5 % |
| NA (abs.; %) | 11 22.6 % | Anthra. (abs.; %) | 108; 22.2 % |
| **Grading** |  | taxane (abs.; %) | 2; 0.4 % |
| G1 (abs.; %) | 43; 8.8 % | CMF (abs.; %) | 92; 18.9 % |
| G2 (abs.; %) | 212; 43.6 % | other (abs.; %) | 68; 14.0 % |
| G3 (abs.; %) | 210; 43.2 % | NA (abs.; %) | 5; 1.0 % |
| NA (abs.; %) | 21; 4.4 % | **HT scheme** |  |
| **HER2** |  | Absent (abs.; %) | 144; 29.6 % |
| Negative (abs.; %) | 298; 61.3 % | Tamoxifen (abs.; %) | 23; 4.8 % |
| Positive (abs.; %) | 80; 16.5 % | LHRHa (abs.; %) | 4; 0.8 % |
| NA (abs.; %) | 108; 22.2 % | Tamoxifen + LHRHa (abs.; %) | 78; 16.0 % |
| **HER2/neu+** |  | AI (abs.; %) | 150; 30.9 % |
| 0 (abs.; %) | 124; 25.5 % | Tamoxifen + AI (abs.; %) | 24; 5.0 % |
| 1 (abs.; %) | 99; 20.4 % | LHRHa + AI (abs.; %) | 13; 2.7 % |
| 2 (abs.; %) | 61; 12.6 % | other (abs.; %) | 44; 9.0 % |
| 3 (abs.; %) | 66; 13.6 % | NA (abs.; %) | 6; 1.2 % |
| NA (abs.; %) | 136; 27.9 % | **Therapy combination** |  |
| **In Situ Component** |  | No (abs.; %) | 4; 0.8 % |
| Absent (abs.; %) | 372; 76.6 % | HT (abs.; %) | 132; 27.1 % |
| G1 (abs.; %) | 17; 3.5 % | CT (abs.; %) | 116; 23.9 % |
| G2 (abs.; %) | 12; 2.5 % | CT + HT (abs.; %) | 169; 34.8 % |
| G3 (abs.; %) | 14; 2.9 % | CT + trastuzumab (abs.; %) | 24; 5.0 % |
| present, not typed (abs.; %) | 69; 14.1 % | CT + HT + trastuzumab (abs.; %) | 39; 8.0 % |
| NA (abs.; %) | 2; 0.4 % | NA (abs.; %) | 2; 0.4 % |
| **Lymphovascular Invasion** |  | **CT months** |  |
| Absent (abs.; %) | 311; 67.0 % | Median; [$q_{1},$ $q_{3}$] | 3; [0, 5] |
| Focal (abs.; %) | 91; 18.6 % | NA (abs.; %) | 14; 2.9% |
| Extensive (abs.; %) | 24; 5.0 % | **Diag. – surg. months** |  |
| present, not typed (abs.; %) | 60; 12.4 % | Median; [$q_{1},$ $q_{3}$] | 0 [0,0] |
| **Lymph Node Stage** |  | NA (abs.; %) | - |
| N0 (abs.; %) | 249; 51.2 % | **Surg. – ther. months** |  |
| N1 (abs.; %) | 172; 35.4 % | Median; [$q_{1},$ $q_{3}$] | 1; [1, 1] |
|  | *(Continued)* | NA (abs.; %) | 18; 3.7% |

**Table S2. Classifiers performance after the iterative consensus procedure for the 5-year and 10-year IDE predictive models.**

|  | **Classifier** | **AUC** (%) | **Acc.** (%) | **Sens.** (%) | **Spec.** (%) | **F1** (%) |
| --- | --- | --- | --- | --- | --- | --- |
| 5-year follow-up | SVM | 90.2 $\pm$ 0.2 | 83.4 $\pm$ 0.2 | 77.0 $\pm$ 0.7 | 85.4 $\pm$ 0.3 | 68.6 $\pm$ 0.4 |
|  | XGB | 93.7 $\pm$ 0.1 | 88.2 $\pm$ 0.2 | 78.1 $\pm$ 0.6 | 91.4 $\pm$ 0.2 | 75.9 $\pm$ 0.4 |
|  | RF | 91.6 $\pm$ 0.1 | 83.9 $\pm$ 0.3 | 80.6 $\pm$ 0.7 | 85.0 $\pm$ 0.3 | 70.4 $\pm$ 0.4 |
|  | NB | 85.1 $\pm$ 0.2 | 81.3 $\pm$ 0.1 | 59.0 $\pm$ 0.4 | 88.2 $\pm$ 0.2 | 59.8 $\pm$ 0.3 |
| 10-year follow-up | SVM | 88.6 $\pm$ 0.2 | 81.3 $\pm$ 0.4 | 76.4 $\pm$ 0.6 | 85.0 $\pm$ 0.5 | 77.6 $\pm$ 0.5 |
|  | XGB | 91.7 $\pm$ 0.1 | 83.3 $\pm$ 0.2 | 78.2 $\pm$ 0.4 | 87.1 $\pm$ 0.3 | 79.9 $\pm$ 0.3 |
|  | RF | 90.0 $\pm$ 0.2 | 82.0 $\pm$ 0.2 | 73.4 $\pm$ 0.4 | 88.4 $\pm$ 0.4 | 77.6 $\pm$ 0.3 |
|  | NB | 84.5 $\pm$ 0.4 | 74.6 $\pm$ 0.4 | 51.6 $\pm$ 0.7 | 91.6 $\pm$ 0.7 | 63.3 $\pm$ 0.6 |
